# Health-Related Quality of Life Is Severely Affected in Primary Orthostatic Tremor

**DOI:** 10.3389/fneur.2017.00747

**Published:** 2018-01-15

**Authors:** Lucie Maugest, Eavan M. McGovern, Katia Mazalovic, Mohamed Doulazmi, Emmanuelle Apartis, Mathieu Anheim, Frédéric Bourdain, Eve Benchetrit, Virginie Czernecki, Emmanuel Broussolle, Cecilia Bonnet, Bruno Falissard, Marjan Jahanshahi, Marie Vidailhet, Emmanuel Roze

**Affiliations:** ^1^Département de Neurologie, EA 4184, Hôpital universitaire de Dijon, Dijon, France; ^2^Department of Neurology, St Vincent’s University Hospital, Dublin, Ireland; ^3^School of Medicine and Medical Sciences, University College Dublin, Dublin, Ireland; ^4^Département de Médecine générale, Faculté de Médecine, Université de Bourgogne, Dijon, France; ^5^Sorbonne Universités, UPMC Univ Paris 06, UMR8256, INSERM, CNRS, Institut de Biologie Paris Seine, Adaptation Biologique et Vieillissement, Paris, France; ^6^Département de Neurophysiologie, Hôpital de Saint-Antoine, Assistance Publique-Hôpitaux de Paris, Paris, France; ^7^Département de Neurologie, Hôpitaux Universitaires de Strasbourg, Hôpital de Hautepierre, Strasbourg, France; ^8^Institut de Génétique et de Biologie Moléculaire et Cellulaire (IGBMC), Illkirch-Graffenstaden, France; ^9^Université de Strasbourg, Fédération de Médecine Translationnelle de Strasbourg (FMTS), Strasbourg, France; ^10^Département de Neurologie, Centre médico-chirurgical Foch, Suresnes, France; ^11^Département de Neurologie, Groupe Hospitalier Pitié-Salpêtrière, Assistance Publique-Hôpitaux de Paris, Paris, France; ^12^Département de Neurologie, Service de Mouvements anormaux, Hôpital Neurologique et Neurochirurgical Pierre Wertheimer, Hospices Civils de Lyon, Lyon, France; ^13^Université de Lyon, Université Lyon I, Faculté de Médecine Lyon Sud Charles Mérieux, Institut des Sciences Cognitives Marc Jeannerod, CNRS UMR 5229, Lyon, France; ^14^CESP, Univ. Paris-Sud, Université Paris-Saclay, UVSQ, INSERM U1178, Paris, France; ^15^Sobell Department of Motor Neuroscience and Movement Disorders, Institute of Neurology, The National Hospital for Neurology and Neurosurgery, University College London, London, United Kingdom; ^16^UMR S 975, CNRS UMR 7225, ICM, Sorbonne Universités, UPMC Université Paris, Paris, France

**Keywords:** primary orthostatic tremor, movement disorder, progressive, health-related quality of life, mixed-method methodology

## Abstract

**Background:**

Primary orthostatic tremor (POT) is a movement disorder characterized by unsteadiness upon standing still due to a tremor affecting the legs. It is a gradually progressive condition with limited treatment options. Impairments in health-related quality of life (HQoL) seem to far exceed the physical disability associated with the condition.

**Methods:**

A multi-center, mixed-methodology study was undertaken to investigate 40 consecutive patients presenting with POT to four movement disorder centers in France. HQoL was investigated using eight quantitative scales and a qualitative study which employed semi-structured interviews. Qualitative data were analyzed with a combination of grounded-theory approach.

**Results:**

Our results confirm that HQoL in POT is severely affected. Fear of falling was identified as the main predictor of HQoL. The qualitative arm of our study explored our initial results in greater depth and uncovered themes not identified by the quantitative approach.

**Conclusion:**

Our results illustrate the huge potential of mixed methodology in identifying issues influencing HQoL in POT. Our work paves the way for enhanced patient care and improved HQoL in POT and is paradigmatic of this modern approach for investigating HQoL issues in chronic neurological disorders.

## Introduction

Primary orthostatic tremor (POT) is a clinical–neurophysiological syndrome characterized by unsteadiness upon standing still due to a high frequency (13–18 Hz) tremor affecting the legs (1). The underlying pathogenesis of POT is incompletely understood but dysfunction of the cerebello-thalamo-cortical motor pathway is thought to play a key role (2–6). POT is a progressive disorder and its pharmacological and surgical treatments are often disappointing, resulting in a gradual and slow worsening of a patient’s condition over the course of the disease (7–9).

While demographics and clinical features of POT patients have previously been reported (8, 10, 11), only one study addressed health-related quality of life (HQoL). This small pilot study suggested both HQoL and mood were impaired in POT, but little is known about what causes these impairments (12). In our clinical experience, fear of falling is a widespread complaint in POT patients. However, its characteristics and consequences have not previously been investigated.

To investigate HQoL in POT, the aim of this study was to identify and explore factors which determine HQoL in our patient series. By exploring what specific aspects of HQoL are affected by POT through a quantitative and qualitative approach, we aimed to tailor our patient’s clinical care. Specifically, we aimed to actively address the HQoL issues highlighted by our patients in the outpatient setting and put in place therapies and social supports which might address these issues. To achieve this aim, we enrolled consecutive POT patients presenting to neurological movement disorder clinics from four French University hospitals. A mixed-methodology approach was employed. Standardized patient questionnaires, with a special focus on fear of falling, were used as an initial quantitative assessment tool. Semi-structured interviews explored issues relating to HQoL in greater depth using a combination of grounded-theory approach and thematic analysis.

## Materials and Methods

### Participants and Study Design

We prospectively enrolled consecutive POT patients seen in movement disorders clinics from four French University Teaching Hospitals (Paris, Lyon, Strasbourg, and Dijon) between March 2014 and December 2015. We included patients with an established diagnosis of POT based on clinical observation and confirmed by a neurophysiological recording showing a 14- to 18-Hz pure orthostatic tremor. Patients with secondary orthostatic tremor or an associated neurological disorder were excluded. All patients underwent a standardized neurological history and examination. In addition, a set of quantitative scales investigating the consequence of their disease on daily life was administered. Finally, a subsample of patients participated in a qualitative investigation aimed at getting in-depth information from the patient’s perspective.

### Data Collection

To assess HQoL in POT, we used the short-form (36) health survey (SF-36) a well-validated assessment tool (13–15) which measures eight domains of health and well-being [physical functioning (PF), activities, limitations, pain, general health (GH), vitality (VT), social functioning (SF), emotional limitations, and mental health]. Scores range from 0 to 100; higher scores indicate better health status and a summed score reflects global HQoL. SF-36 scores from POT patients were compared to published scores for the general population (90), and PD (16).

Fear of falling in POT patients was investigated using three validated scales (17): the survey of activities and fear of falling in the elderly (SAFFE), the falls efficacy scale (FES), and the consequences of falling (COF) (18–20). Different versions of these scales exist with minor differences in the scoring method. To allow further comparison with literature data, we used versions of the SAFFE (21), COF (17, 19), and FES (22) that were previously used to study series of healthy subjects and PD patients. The SAFFE rates a patient’s likelihood of avoiding 17 activities (1 = never avoid to 3 = always avoid); the total score ranges from 0 to 34, with higher scores indicating higher activities restriction. The FES measures a patient’s perceived efficacy at performing 10 activities of daily life without falling; the total score ranges from 0 (high fear of falling) to 100 (no fear of falling). The COF rates a patient’s concern about falling in 12 questions; the total score ranges from 12 to 48, with higher scores indicating a greater concern about falling. Two subscores can be calculated: damage to identity (COF-DI) and functional independence (COF-LFI). Fear of falling scores from POT patients were compared with available scores for the general elderly population (19, 20) and Parkinson’s disease (PD) (17, 21).

Depression and anxiety were evaluated using the hospital anxiety and depression scale (HADS) (23), a 14-item questionnaire which calculates two scores—one for depression (HADS-D) and one for anxiety (HADS-A). The total score ranges from 0 (normal) to 21 (severe disorder) for both HADS-D and HADS-A with a cutoff point <8 (23, 24).

To assess coping we used the stigma scale (25), the acceptance of illness scale (AIS) (26), and the Rosenberg’s self-esteem scale (RSES) (27, 28). The stigma scale assesses perceived stigma in six items relating to self-image and relationship with others; summed score range from 0 to 18, a score higher than 7 reflects moderate stigma and higher than 12 reflecting severe stigma. AIS is an eight-item scale; each item is rated from 0 to 4, with a total score of 40. Higher score reflects better acceptance and score below 30 reflects poor acceptance. RSES is a 10-item scale assessing individual’s feeling of self-worth in a five-point (0–4) Likert scale. The total score ranges from 0 to 40—a score below 25 reflects a low self-esteem.

Purposive sampling was used for the qualitative study. This method of sampling includes patients based on their individual characteristics (age, sex, disease duration, and disability level) to achieve the maximum diversity of opinion and representation. A semi-structured interview guide was developed in a step-wise manner, by two investigators. The investigators were a neurologist, specialized in movement disorders and a general practitioner trained in qualitative research without a specialized knowledge of neurology. These two complementary investigators made an overall assessment of the impact of POT in everyday life in a clinical and pragmatic way. Recruitment continued until saturation in data analysis was reached. Face-to-face, in-depth, semi-structured interviews with participants were performed in a hospital office but independent of any context of care. Interviews focused on how patients experienced the impact of POT on their daily life, with open-ended questions concerning broad areas in relation to the disease, including history of the disease and the diagnosis, symptoms and handicap related to the disease, effects of the treatments, psychological impact and social life, and their knowledge or beliefs about the disease. Interviewers reworded or clarified the question where appropriate to further investigate topics brought up by participants. Field notes were taken before and during the interview to identify contextual details and non-verbal expressions. Each interview lasted between 30 and 60 min and was digitally recorded and transcribed verbatim.

### Data Analysis

#### Statistics

All manipulations and statistical analysis were implemented with R (3.3.0). Data were inspected for normality and transformed if necessary. Comparison of mean scores from scales assessing fear of falling with the published data was assessed using a two-tailed Student’s *t*-test.

Descriptive socioeconomic data are presented as mean ± SD of each scale score. Clinical characteristics are described in numbers and percentages. As the literature review provided little or no information about our population parameters necessary for sample size planning, we performed the *post hoc* power analysis (95–100%) in some significant variables (between POT and age-matched healthy subject).

Data were inspected for normality using the Kolmogorov–Smirnov test. Pearson correlation coefficients selected as the assumptions of normality were not violated to calculate the direction and magnitude of association between variables.

Associations between study-specific factors with SF-36, COF, FES, or SAFFE were analyzed using multiple linear regression. To determine the relative importance (RI) of variables (29, 30), we used the Linderman, Merenda and Gold (LMG) method with the R-package RELAIMPO (31). This method allows the contribution of different correlated regressors to be differentiated in a multiple linear regression model. This metric is based on unweighted averages over sequential *R*^2^’s of each variable in all permutation of available regressors to avoid regressors’ order effects. The total *R*^2^ is split into one non-negative *R*^2^ share per regressor, all of which sum to the total explained *R*^2^. This is done by calculating the contribution of each predictor at all possible points of entry into the model, and taking the average of those contributions. RI estimates are then adjusted to sum to 100% for easier interpretation. Confidence interval estimates of the RI coefficients indicating whether regressors differed significantly from each other in their RI contributions were obtained using the bootstrapping capabilities of the RELAIMPO package; a *p* value of <0.05 was used as a cutoff for statistical significance.

#### Qualitative Analysis of the Interviews

Data analysis was conducted according to the principle of grounded theory following an inductive method that allows themes and patterns to emerge from the data without any *a priori* hypotheses (32). The data were analyzed by two individuals with secondary comparisons of their analysis and discussion of differences to ensure inter-case consistency. Both researchers approached data analysis without any pre-established hypothesis while remaining open to the study objective. Researchers were carefully selected to ensure a complimentary but different profile and training: a neurologist familiar with movement disorders and a general practitioner trained to qualitative research. This selection process of complementarity prevents interpretation bias as fully as possible. The major themes were identified from comparing, contrasting, and categorizing the codes. Both investigators used N’Vivo software for storage, searching, and coding qualitative data. Data saturation was discussed between the two interviewers.

### Study Approval

The local ethics committee (CPP Ile de France 6, Salpêtrière Hospital, Paris) reviewed and approved the study protocol. The study was classified as an observational study, and the data collection was then approved by the national commission for data protection (CNIL—number of approval: 1975100) according to the rules of the French regulation. As local ethic committee did not constrained to get written consent for this observational study, oral informed consent was obtained from all participants after detailed verbal explanations, and the study was performed according to the approved protocol.

## Results

We enrolled 40 consecutive patients with POT from four centers in France. Demographic characteristics of patients are summarized in Table 1.

**Table 1:** Clinical and demographic characteristics of 40 POT patients.

	Mean	SD
Age (years)	68.51	8.27
Sex ratio (M:F)	1:5	
Age at onset (years)	52.54	11.06
Disease duration (years)	15.96	10.85
Time to diagnosis (years)	9.21	8.75
Tremor frequency (Hz)	16	1.16

*POT, primary orthostatic tremor; M, male; F, female*.

### Evaluation of the HQoL

Detailed quantitative evaluation of their HQoL is shown in Table 2.

**Table 2:** Evaluation of health-related quality of life.

	Mean	SD
**Quality of life**		
SF-36—total (0–100)	53.8	16.91
SF-36—physical health (0–100)	37.19	9.46
SF-36—mental health (0–100)	47.73	8.95

**Fear of falling**		
SAFFE (17–51)	28.74	6.73
FES (0–100)	71.44	19.97
COF (0–48)	30.36	8.41
COF_DI	16.21	4.38
COF_LFI	14.1	4.77

**Depression and anxiety**		
HAD_depression (0–21; *N* < 8)	5.667	3.33
HAD_anxiety (0–21; *N* < 8)	8	4.81

**Coping**		
AIS (0–40; *N* > 29)	23.13	8.49
Stigma scale (0–18; *N* = 0)	7.25	4.44
RSES (0–40; 24 < *N* < 36)	33.26	4.67

*SAFFE, survey of activities and fear of falling in the elderly; FES, falls efficacy scale; COF, concern of falling scale; AIS, acceptance of illness scale; RSES, Rosenberg’s self-esteem scale; HAD, hospital anxiety and depression; SF-36, short-form (36) health survey*.

**Figure 1:**
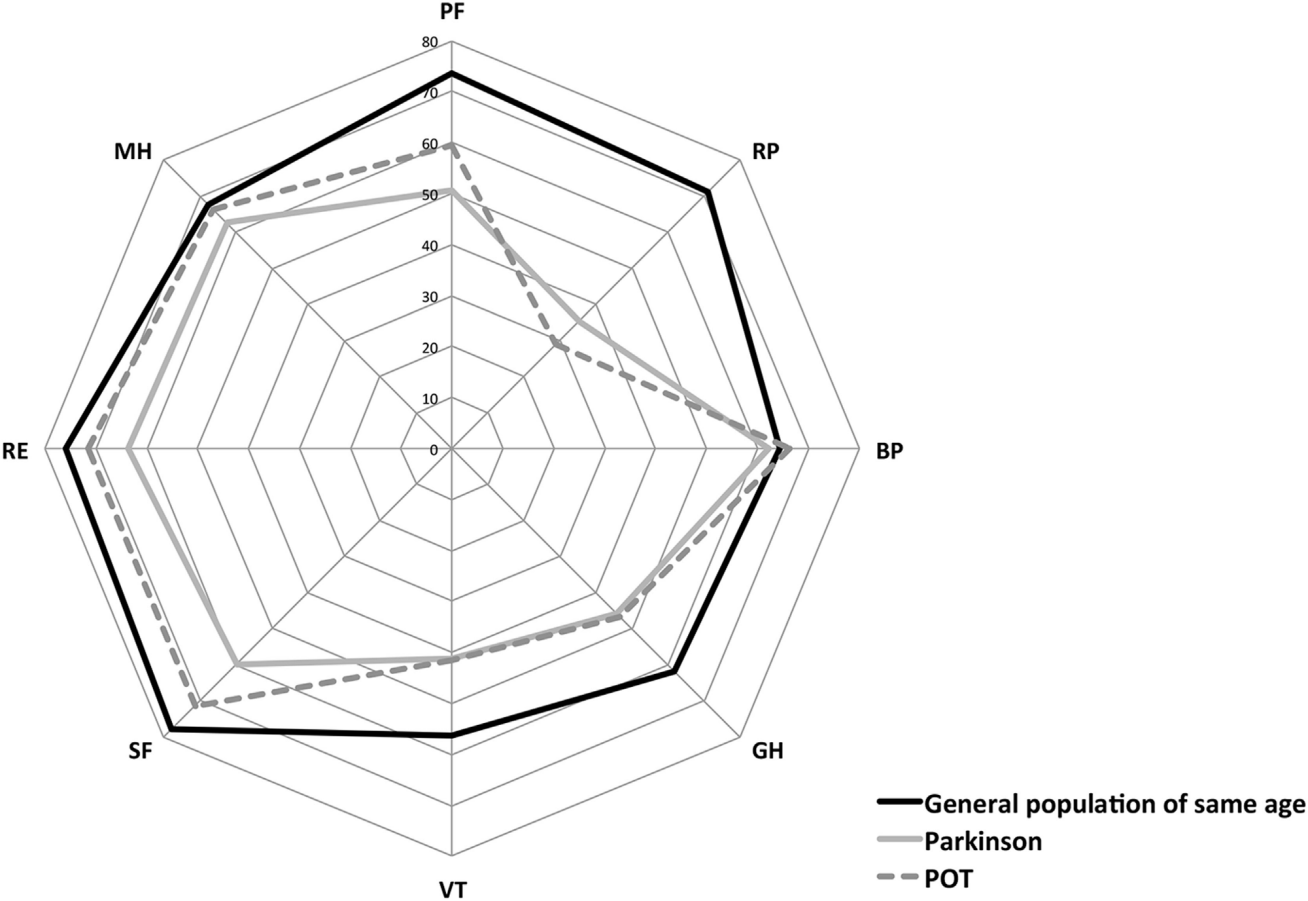
Comparison of health-related quality of life of Primary Orthostatic Tremor (POT), patients with age-matched healthy subjects and patients with Parkinson’s disease. Values are shown for each dimension using chart polar plots: physical functioning (PF), role limited by physical problems (RP), bodily pain (BP), general health (GH), vitality (VT), social functioning (SF), role limited by emotional problems (RE), and mental health (MH). SF-36, the short-form (36) health survey; POT, primary orthostatic tremor.

Lower scores on the SF-36 reflect poorer HQoL. A comparison of SF-36 scores with data from the general French population of a similar age (14) showed lower scores on all eight domains of the scale, particularly in PF, activities limitations, GH, and VT. Although direct statistical comparison with a previous study was not possible, POT patients appeared to have scores similar to patients with PD (33), with the exception of scores for physical and SF and emotional limitations, which seemed slightly better (Figure 1).

Fear of falling aligned with results from the PD literature. The mean SAFFE score was 29.21. This is similar to scores obtained in PD patients of the same age in two independent studies (mean = 27.2 after conversion for different scoring, SD = 6.95; *N* = 102; *p* = 0.19) (17) (mean = 26.2; SD = 8.40; *N* = 71; *p* = 0.07) (21). Moreover, these results are greater than what was observed in older subjects in the general population, meaning that POT patients and PD patients are more prone to avoid activities due to fear of falling than older healthy controls (mean = 24; SD = 6.8; *N* = 224; *p* < 0.001; mean age = 80.7; *p* < 0.0001) (19). The mean FES score was 71.44. Studies of healthy controls reported greater self-efficacy (mean = 84.9; SD = 20.5; *N* = 1103; *p* < 0.001), despite sampling an older population (mean age = 79.6; SD = 5.2; *N* = 1103; *p* < 0.001) (22). The mean COF score was 30.36, with a significant higher “damage to identity” (DI) subscore (mean = 16.21; SD = 4.38) than the “loss of functional independence” subscore (mean = 14.1; SD = 4.77; *p* = 0.045). Mean COF score in PD patients from Rahman et al. was significantly lower (mean = 26.9; SD = 7.90; *N* = 110; *p* = 0.03), reflecting a greater concern about falls in POT patients than in PD patients. There are few studies of COF in healthy subjects, but Yardley and Smith founded similar perceived COF although their population was clearly from an older cohort (mean = 26.75; SD = 7.8; *N* = 126; *p* = 0.02) (19).

As assessed by the HADS, 18% of patients had moderate-to-severe depression and 42% had anxiety. Patients had difficulties accepting their disease and adjusting to their diagnosis, as reflected by the low mean score on the AIS. The mean perceived stigmatization was moderate, which included 8% of patients with severe perceived stigmatization and 8% that perceived no stigmatization. Self-esteem was not affected by the disorder.

### Intercorrelations between Psychosocial and Clinical Variables and HQoL

Short-form (36) health survey scores correlated (*p* < 0.01) with disease duration, gender, and with psychosocial variables in the following direction: positively with perceived efficacy in performing daily living activities without falling (β = 0.69—FES), self-esteem (β = 0.43), and acceptance of illness (β = 0.33), and negatively with concern about falls (β = −0.66), restriction of activity (β = −0.58), perceived stigmatization (β = −0.57), depression (β = −0.53), and anxiety (β = −0.42).

Similarly, depression correlated with fall efficacy (β = −0.51, *p* < 0.01), restriction activity (β = 0.47, *p* < 0.01), perceived stigmatization (β = 0.50, *p* < 0.01), and self-esteem (β = −0.44, *p* < 0.01), whereas anxiety correlated (*p* < 0.01) with concern about falls (β = 0.58), fall efficacy (β = −0.36, *p* < 0.01), and stigmatization (β = 0.47, *p* < 0.05).

### Predictors of HQoL, Depression, and Fear of Falling

**Figure 2:**
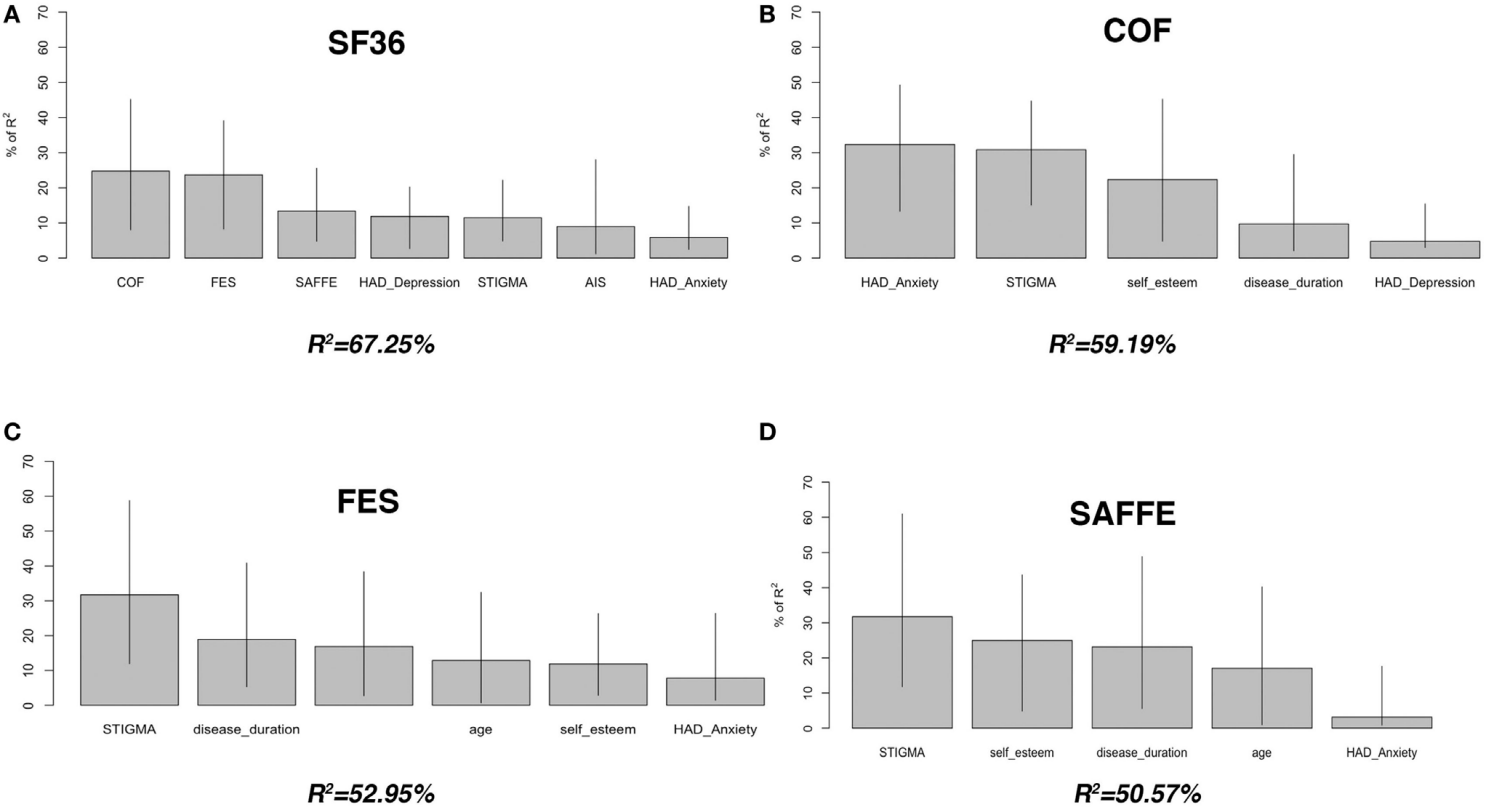
Contributors of health-related quality of life and fear of falling in patients with primary orthostatic tremor. We used Lindeman, Merenda and Gold (LMG) *R*^2^ to estimate the relative importance of each regressor based on variance decomposition of variables included in the final models. The barplots show the proportion of variance in the response variable explained by each explanatory variable, with bootstrapped 95% confidence intervals. The scores were derived from the overall significant *F*-test in a one-way analysis of variance, i.e., *F*_(7, 31)_ = 9.096, *p* < 0.0001, *R*^2^ = 67.25 **(A)**, *F*_(5, 33)_ = 9.57, *p* < 0.0001, *R*^2^ = 59.19 **(B)**, *F*_(6, 32)_ = 6.00, *p* < 0.0001, *R*^2^ = 52.95 **(C)**, and *F*_(5, 33)_ = 6.78, *p* < 0.0001, *R*^2^ = 50.57 **(D)**. SF-36, short-form (36) health survey; COF, concern of falling scale; FES, falls efficacy scale; SAFFE, survey of activities and fear of falling in the elderly.

To determine which factors had the greatest contribution to HQoL scores, the variables correlating with HQoL were used in a multiple regression analysis with HQoL as the dependent variable. To adjust for possible correlations between variables, the RI assessment based on variance decomposition (*R*^2^) was conducted by calculating the relative contribution of each factor. Figure 2 shows the RI of all predictors included in the final models, i.e., the proportion of variance in the response variable explained by each predictor.

Fear of falling was the major predictor of HQoL, as items related to fear of falling accounted, respectively, for 25% (COF), 24% (FES), and 14% (SAFFE) of the variance of SF-36 score (Figure 2). Depression and stigma were other significant predictors, accounting for a further 12 and 11% of the variance.

We used the same method to isolate the contributors of fear of falling with successively FES, SAFFE, and COF as the dependent variable. Perceived stigmatization was the main predictor, accounting for 31.7% of the variance of FES and SAFFE, and 30.8% of the variance of COF. Other predictors of fear of falling are shown in Figure 2.

### Qualitative Data: Themes That Emerged from the Semi-Structured Interviews

**Figure 3:**
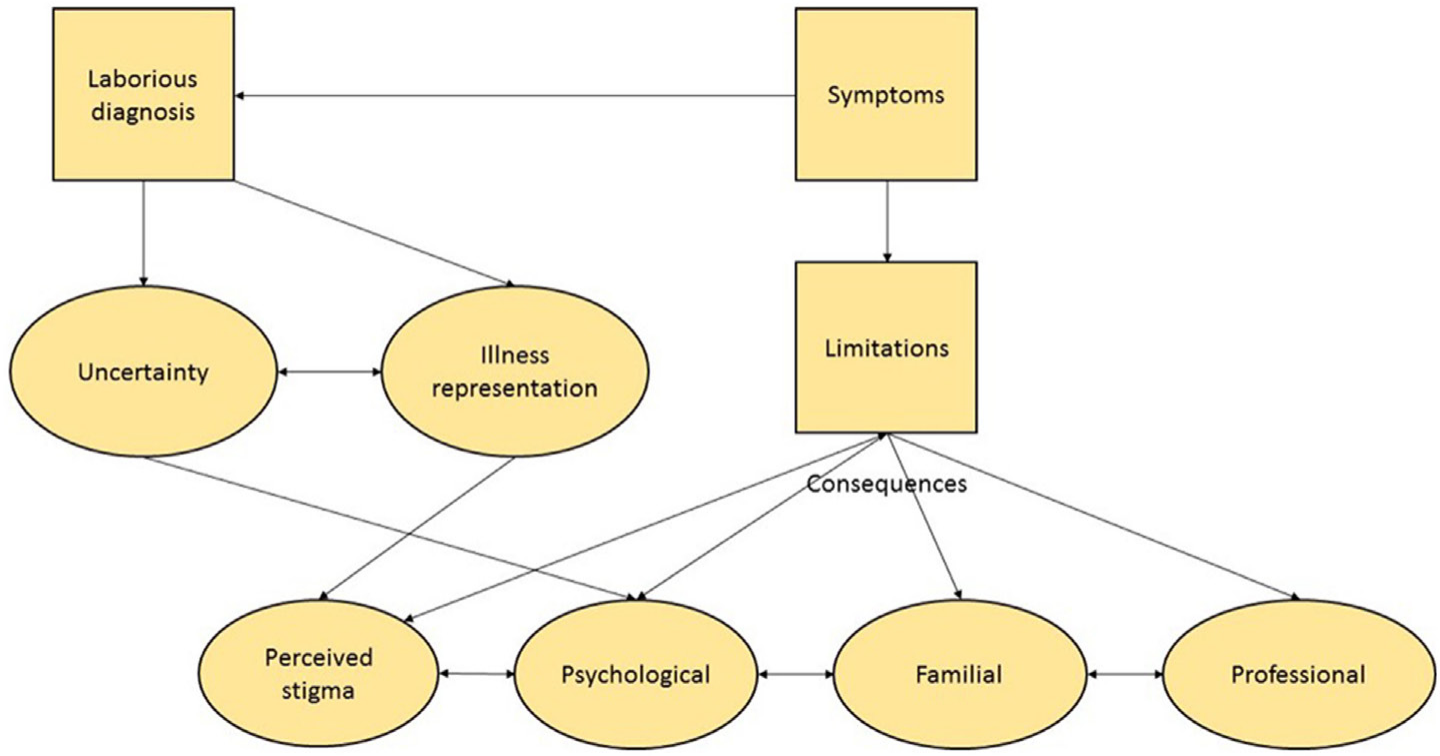
Grounded-theory model showing the impact of primary orthostatic tremor (POT) on daily life based on interviews. The flowchart was derived using a grounded-theory approach for the analysis of transcribed patient interviews. Two researchers with complementary profiles extracted the meaning of the patient’s spoken language by using a constant comparative method and grouping the data into themes and categories. Categories were subsequently organized to establish connections between the various codes, themes, and categories. The data which emerged illustrate the impact of POT on a patient’s everyday life. The directionality of the arrows demonstrates the interplay between each of these elements.

A conceptual framework illustrating interrelationship between the major themes identified from the data is shown in Figure 3.

#### Activities and Social Life Limitations

Most patients reported that their condition impacted on their daily life which is illustrated by this patient’s comment: “*I can do the household chores but from a seated position. I have a rolling stool so that I can vacuum, mop the floor, dust: as long as I sit, no problem. I even did some painting!*”; another patient adapted her bathroom for enhanced autonomy: “*In the bathroom I have a wheelchair and a stool in the shower for washing, [laughing], I laugh but it is annoying*.” Despite these adaptations, patients neglected certain activities, particularly those requiring them to stand still.

These limitations also impacted a patient’s professional life (professional redeployment, disability pension) and social interactions (restriction of social contacts, including contacts with family members). This was not only linked to disease severity but also to an individual’s resources and coping strategies as illustrated by this patient’s comment: “*Previously when dealing with computer troubleshooting, I used to go to customers to carry out repairs—I had to carry the equipment and to go under the desk to make connections. So, I had to change my job so that I conduct troubleshooting just on the phone*.”

#### Illness Representation and Perceived Stigmatization

In most patients, the diagnosis was delayed which had repercussions for their psychological condition. One patient explained, “*My GP told me, when I explained I was shaking occasionally, he told me stuff that is said to everyone: You’re crazy, you should see a psychiatrist, you are mad. Okay, I saw a psychiatrist. And then, from doctors to psychiatrists, [*…*], my journey was quite difficult*.”

Patients perceived their illness as a rare and unknown disease which significantly contributed to disease disability. The reality of daily life belied the actual disability. Social pressure emerged as a frequent theme: fear of looking drunk, or being accused of simulating or seeking secondary benefits. Others tried to rationalize their symptoms in the context of other disease as illustrated by this patient’s comment: “*It’s plenty of little things that, put all together, well we realize that… well there is worse eh, other diseases… god knows that we can see cancers, things like that which are unbearable, but here it is, here it is… at least someone who has cancer, it’s apparent people will take care of that person but me… I always have to explain myself… except with the people who know me, at who know what I can do or not…*.” The same patient, rationalizes later her remarks by quoting her neurologist: “*Pr X said things clearly at the outset: it won’t kill me, but here it is not an illness with vital organs damage, but it will rot your life, and indeed*….”

#### Vision for the Future

The burden of uncertainty pertaining to the limited available scientific knowledge was identified as an important theme. Most patients reported a prominent fear of falling, even when they had never fallen. Fears about their future was also frequently reported. Such fears were fueled by the perceived gradual worsening of their condition, their functional limitations, the rarity of the disease, and the poorly explained pathophysiology as illustrated by the following comment:

*“This disease that no one knows… I was not sure where it was going… how it would evolve; we still do not know*. *How much time we’ll really be able to… stand upright or is it a moment we’re going to be forced to… use a wheelchair, to be clear. Well, it’s true that psychologically it’s hard …”*; *“Eventually, you’re looking ahead to the future and asking yourself, ‘Am I going to finish in a wheelchair or to become highly dependent on others’ and that’s something you may be worried about. What would bother me the most is being very dependent on others, that is something I will bear very badly.”*; "*That means that I think for myself: let’s hope that no harm comes to my husband because what could I do? Because well [sobs], we have children but they work, you can’t count on children all the time, they have their lives* [sobs].”

## Discussion

Using a novel mixed-methodology approach, we found that HQoL was severely impaired in POT and the most significant factor contributing to it was fear of falling. Furthermore, we identified perceived stigma was the main determinant for fear of falling. Themes identified in the qualitative analysis included: dealing with a virtually invisible yet crippling disease and coping with the uncertainty of a rare disease whose pathophysiology is unknown. These factors fueled concerns about the future and thus influenced HQoL. Our findings, which enhance our understanding of POT-related disability, will help to develop more tailored treatment programs for patients.

### Fear of Falling Strongest Predictor of HQoL

Quantitative data demonstrated that HQoL was markedly impaired across all domains in patients with POT—in the same range as observed in PD (33). Our results corroborated findings from an earlier study which examined HQoL parameters in a smaller number of POT patients (12). We examined a broad range of factors to identify which impacted most on HQoL in POT. Fear of falling was consistently identified as the main contributor in POT patients, irrespective of the investigative tool used. The fear of falling belies the actual incidence of falls among this patient cohort (34). Restriction of activities, concern about falling and poor perceived self-efficacy relating to fear of falling were identified as the strongest predictors of HQoL. These three components of fear of falling have previously been shown to reflect independent psychological processes with varied consequences on daily life (17, 35). Fear of falling is well known to be a strong predictor of impairment of HQoL, in the elderly (36) and in PD (17, 37). However, unlike PD where fear of falling is coupled with a high incidence of falls (38, 39) POT patients rarely fall. Interestingly, after accounting for disease severity, fear of falling maintained a strong association with impairment of HQoL whereas actual falls did not (37). Among the epidemiological factors we assessed, only disease duration correlated significantly with poor HQoL, a greater fear of falling and increased incidence of depression. Fear of falling has been successfully managed in PD and elderly cohorts using a multidisciplinary approach involving cognitive behavioral techniques and exercise therapy (17, 40, 41). These techniques could be applied to patient with POT to combat fear of falling and improve HQoL.

### The Impact of Depression, Anxiety, and Perceived Stigmatization on Patient HQoL

Despite a low incidence of depression among our cohort (18%), it was identified as the fourth most significant predictor of HQoL in POT. Furthermore, depression was strongly associated with a low perceived self-efficacy relating to falls. Depression’s effect on HQoL has been well described across a variety of chronic diseases (12, 33, 42, 43). Anxiety, which was reported in 42% of our patients, was also significantly associated with impaired HQoL. It was the strongest predictor of concerns about falling and an independent predictor of activity restriction related to fear of falling. Our findings are similar to what has been observed in PD, in which depression and anxiety have been identified as independent predictors of fear of falling (17). However, contrary to PD, where the neuropsychiatric symptoms are part of the non-motor syndrome linked to the underlying neuropathology (44–46), these symptoms are less well understood in POT and might be more related to the particular burden of the disease in daily life. We therefore employed a qualitative approach to explore these and other symptoms linked to POT-related disability further. Physical limitations and related professional and familial consequences, fear of the future, uncertainty surrounding the disease and perceived stigmatization were prominent themes in the qualitative study. This suggests these issues play a key role in the psychological burden associated with POT. Fear relating to a specific illness and its future have been shown to significantly contribute to patient morbidity in rare and in chronic disease (47–49). It has also been established that the neural circuitry underlying anxiety is intrinsically linked to uncertainty (50). Therefore, patients with disease-related anxiety, like those with POT, may benefit from therapeutic interventions which emphasize accurate predictions of their future, dispel potential misconceptions, and enhance coping strategies for threat uncertainty (50).

Perceived stigmatization was identified as a significant predictor of HQoL and the major contributor to fear of falling in POT. Our qualitative evaluation explored this issue in depth and identified multiple processes influencing perceptions of stigma among patients with POT. These included a delay to diagnoses or misdiagnosis and a feeling that their disease was rare and unknown. Furthermore, a fear of how others perceived them and a lack of clinically obvious features on their exam compounded feelings of stigmatization in our patient cohort. Stigma is defined as an attribute that is deeply discrediting and reduces someone from a whole and usual person to a tainted discounted one (32). Perceived stigma, on the other hand, is an awareness of social devaluation or discrimination based on a socially stigmatizing attribute and has a stronger and more direct relationship with HQoL (51). It is frequently internalized by individuals and not addressed by health-care professionals (52). It has been previously shown to have far-reaching negative consequences for people with chronic illness including reduced HQoL, greater functional impairment, relationship difficulties, and social isolation (25, 53–58). As such, it is an important consideration when treating patients with chronic conditions, and it has recently been recognized as an emerging public health concern (59). Patients with rare diseases such as POT are particularly susceptible to perceived stigmatization as patient education and public awareness is often limited in such rare conditions (60–63). In our series, patient distress was significantly compounded by lack of information about their diagnosis both in the public and medical domain which led to concerns about their prognosis. Our findings have significant implications for clinical practice as they provide practical applications for the consultation. They prompt us (i) to address the issue of perceived stigmatization and to discuss coping strategies with our patients and (ii) to clearly outline the current medical knowledge available on POT. It has been shown that chronic conditions that employ patient education programs on issues such as stigma have positive outcomes with regard to patient coping strategies and HQoL (64–67). Similarly, interventions used in mental illness that specifically target perceived stigma have been shown to increase self-esteem and self-efficacy (68). Such interventions could be adapted for patients with POT to reduce disease-related disability and improve HQoL. In considering our results, a multidisciplinary approach to treatment that goes beyond the biological and physiological consequences of the disease, seems a necessary step moving forward.

### Mixed-Method Study, a Useful Tool to Assess Quality of Life in Chronic Neurological Disorders

In our study, we employed a mixed-methodology approach using both quantitative and qualitative measures to assess HQoL in POT. HQoL is an important clinical outcome that can significantly alter disease-related disability (69, 70). Assessment of this complex area is neither easily quantified nor defined and requires a multidimensional approach (71, 72). Unlike other measurements used in research, HQoL is a uniquely personal perception that cannot be neatly characterized by quantitative data alone (73). Increasingly, it is considered to be an important clinical outcome like other traditional biological end points of disease (74). Questionnaire-derived HQoL scores are a standardized assessment tool that are useful when comparing results across different studies and diseases (75, 76). In comparing our results with those from PD studies, we identified similar impairments in HQoL despite differences in physical disability and prognosis. However, these quantitative measures alone do not provide a comprehensive insight into how chronic disease affects daily life nor do they permit exploration of unexpected themes (77–79). Qualitative research on the other hand focuses on the human experience through systematic and interactive approaches (80, 81). Unlike hypothesis-driven quantitative methods, this approach can capture unanticipated data in a naturally occurring, uncontrolled form. While medical researchers with deep historical roots in quantitative data were initially reluctant to employ qualitative approaches, these methods are being increasingly used in health-care research to capture a patient’s perspective (82–85). In qualitative research, the emphasis is the accuracy and precision of capturing a patient’s point of view rather than reliability or hypothesis testing. In our study, we applied the accepted quality criteria for qualitative research such as purposive sampling, multiple coding, and triangulation to ensure a rigorous, standardized, and systematic approach (86). Qualitative methods have been effectively used to capture the psychosocial burden of disease (65–69). Our qualitative data bolstered results from our quantitative data while highlighting themes not explored in patient questionnaires or anticipated by our researchers. We gain a better understanding of the meaning and implications of the quantitative findings, with a deeper understanding of participants’ opinion, representations, or reasoning. For example, the poor scores on anxiety scales, like what has been observed in PD, a condition which is indisputably “more severe” than POT, could be considered paradoxical. However, when taken together with the qualitative analysis which emphasized the feeling of uncertainty and complexity of illness representation as a source of anxiety in POT patients, we can better interpret our quantitative findings.

Qualitative methods alone has its limitations—it does not allow for comparisons, severity assessment, prevalence estimates, or assessment of efficacy of an intervention procedure (87). A mixed-methodology approach is successful at overcoming these limitations by combining both methods (88–90). Our mixed-method approach reflects a convergent parallel design allowing reciprocal interpretations between qualitative and quantitative data (91). The importance of mixed methodology in health research is increasingly recognized; the proportion of mixed-methodology studies has almost doubled in the last decade (92–96). This method is very much in its infancy in neurology research and has been used to investigate single issues (97–100), caregiver’s burden (101, 102), and prospective studies (103, 104). It is outside the field of neurology where it has been shown to be effective in assessing the more global issue of HQoL (105, 106). Our work is paradigmatic of a mixed-methodology approach that holds huge potential for in-depth investigation of HQoL issues in chronic neurological disorders. Moving forward we believe this comprehensive experimental approach should be the gold standard for clinical studies aimed at understanding issues related to the burden of chronic neurological disease and acceptance of its treatment from a patient’s perspective. In the case of POT, it was crucial to raise awareness on the importance of perceived stigmatization and the anxiety about the future in order to better take care of these patients. Special clinical attention is required to prevent patients from experiencing the additional burden of stigma and its negative psychological consequences. Moreover, a more detailed explanation of a patient’s long-term outcome might reduce the burden of uncertainty and in so doing improve quality of life for POT patients.

### Limitations of Our Study

We acknowledge several limitations in our study. As there was no age-matched healthy control group for our quantitative data, we compared our results on HQoL with published data on an age-matched general French population. Furthermore, fear of falling was compared to published data on PD patients of the same age and with an older general population. As there is no available cutoff values for these scales, this informed us on the severity of fear of falling in our POT population.

Second, due to the lack of a validated quantitative tool the severity of the disorder could not be quantitatively studied. As such, it could not be used in the multivariate analysis, even though it probably has a role in HQoL and in fear of falling. Nevertheless, despite the progressive nature of POT, duration of the disease had no effect on HQoL and fear of falling, suggesting that severity has not a major role.

These limitations are counteracted by the use of a thorough qualitative study, allowing an in-depth study of the burden of POT on daily life.

## Conclusion

Using a mixed-methodology approach, our results illustrate what factors impact HQoL in POT. By identifying issues such as fear of falling, uncertainty about the disorder and its course, perceived stigmatization, and patient-related anxiety, we can tailor our consultation to discuss these issues and take them into account for the overall care recommendation.

## Ethics Statement

The local ethics committee (CPP Ile de France 6, Salpêtrière Hospital, Paris) reviewed and approved the study protocol. The study was classified as an observational study, and the data collection was then approved by the national commission for data protection (CNIL—number of approval: 1975100) according to the rules of the French regulation. Oral informed consent was obtained from all participants after detailed verbal explanations, and the study was performed according to the approved protocol.

## Author Contributions

ER, MV, MJ, BF, and KM designed the study. ER, MV, and KM supervised the study. LM, ER, EM, KM, BF, MJ, and MD drafted/revised the manuscript. LM, MV, KM, MD, EA, MA, FB, EB, VC, CB, EM, and ER acquired and analyzed/interpreted the data.

## Conflict of Interest Statement

The authors declare that the research was conducted in the absence of any commercial or financial relationships that could be construed as a potential conflict of interest.
